# Cases of Intermittent Cured by Calomel

**Published:** 1808-01

**Authors:** Daniel Wilson


*Cases of Intermittent cured by Calomel.* By Daniel Wilson, M. D.—In a Letter to Dr. John Redman Coxe.

I Take the liberty of stating to you, in a cursory manner, a few cases, in which mercury has appeared to be successfully used in the cure of Intermittent Fevers. In

In the Autumn of 1803, I prescribed for a lady who lived in the country. Her case was stated to be by her husband, as a long-protracted intermittent of the quartan type. I directed a cathartic of jalap and calomel, as introductory to the Peruvian bark. A few days afterwards, I received a letter from the husband, expressing much uneasiness for his wife. He stated that her mouth was very sore*, and that he was afraid I had made a mistake, and had given mercury to his wife. I answered his letter, and prescribed a saline cathartic, together with an astringent gargle: a few days afterwards I saw the gentleman, who expressed much satisfaction at the recovery of his wife. He observed to me, that as soon as her mouth became affected, she never had a return of the ague.

Every physician is ready to bear testimony to the efficacy of mercury in those diseases commonly called bilious; and as a variety of the same disease, I readily concluded that in this instance mercury had cured the intermittent.

Until last December a fair opportunity did not offer, by which I might satisfy myself more fully. In that month a poor young man, a journeyman baker, called on me for advice; his case was also a long-protracted quartan. I prescribed mercury for him in such doses as gently to touch his mouth, and had the satisfaction to ascertain that as soon as his mouth became affected, his agues left him. He was extremely reduced by the disease, and had the appearance of a very great predisposition to dropsy. His cure was confirmed by the use of the Peruvian bark.

Within these few days I am favoured with a similar case from a practitioner in the country, which, like my first, was the result of accident. A child, under two years of age, laboured under an intermittent fever: a cathartic, of which calomel composed a part, was prescribed, but not operating, a sore mouth was induced, and the child has not had a return of the ague.

These cases stand too much alone to establish a principle in medicine, but I am very fully persuaded that they may be confirmed by further experience.

Should they be found worthy of a place in your Medical Museum, you are very much at liberty to insert them.

*Richmond, (Virginia) Nov. 3, 1805.*

